# Supplementary material for: Sustained-input switches for transcription factors and microRNAs are central building blocks of eukaryotic gene circuits
Source: Genome Biol. 2013 Aug 23;14(8):R85. doi: 10.1186/gb-2013-14-8-r85 (PMC4054853; doi:10.1186/gb-2013-14-8-r85)
Supplement: Additional file 5 — HTML Browsable Motif Output. Zipped folder containing all WaRSwap and FANMOD motif output, viewable in a web browser. [file gb-2013-14-8-r85-S5.ZIP › HTML_browsable_motif_output/FANMOD_ath_tair9/sigs_fanmodm-2000.pvals.heatmaps.html/motif_id_38_001101002_tftype_ath_upstream_-1000_0.html]

```
BG_MODEL = FANMOD
MOTIF_ID = 38_001101002
TF_TYPE = ath
UPSTREAM = -1000_0


PVals
FN_0.2	FN_0.4	FN_0.6	FN_0.8
dg_60.genes	0.98	0.589	0	1
dg_70.genes	0.986	0.589	0	1
dg_80.genes	0.984	0.582	0.001	1

ZScores
FN_0.2	FN_0.4	FN_0.6	FN_0.8
dg_60.genes	-2.119	-0.212	3.351	-3.564
dg_70.genes	-2.162	-0.221	3.336	-3.594
dg_80.genes	-2.147	-0.224	3.305	-3.487

StDevs
FN_0.2	FN_0.4	FN_0.6	FN_0.8
dg_60.genes	38449.56	27137.233	5329.203	775.131
dg_70.genes	37700.405	26704.528	5351.473	774.887
dg_80.genes	37612.308	26581.667	5351.947	782.136
```
